# Social media usage of chinese nursing students: Attitudes, motivations, mental health problems, and self-disclosure

**DOI:** 10.1371/journal.pone.0277674

**Published:** 2022-12-14

**Authors:** Xinhong Zhu, Chongming Yang, Linlin Ding, Xiaona Zhang, Guiyuan Qiao, Xiaolian Gao, Fen Yang

**Affiliations:** 1 School of Nursing, Hubei University of Chinese Medicine, Wuhan, China; 2 College of Family, Home, and Social Sciences, Brigham Young University, Provo, Utah, United States of America; Northumbria University, UNITED KINGDOM

## Abstract

**Background:**

Excessive self-disclosure online may risk the reputations, mental health problems, and professional lives of nursing students. This study investigated nursing students’ usage of social media, their attitudes towards social media, mental health problems and self-disclosures, and the relationships of these variables.

**Methods:**

A cross-sectional study was conducted online (n = 1054) with questionnaires of Fear of Missing Out (FoMO), Social Media Fatigue (SMF), Students’ Uses and Views of Social Media (SUVSM) and self-disclosure in social media which included self-information shown on social media and information viewed by others.

**Results:**

Although most of them held positive attitudes towards social media, 17.4% of the participants acknowledged that they had posted inappropriate contents online and 37.6% witnessed improper posts from schoolmates or teachers online. SMF was affected by familiar with relevant regulations on the social media usage (*β* = -.10, *p <* .001), FoMO (*β* = .41, *p <* .001), and SUVSM (*β* = .17, *p <* .001). Additionally, nearly 1/3 participants reported their net-friends could view following information: gender, age, occupation, education level and location. Self- disclosure in social media was positively influenced by education (*β* = .10, *p <* .001), sharing moments or Weibo, etc. (*β* = .009, *P* = 0.009), time spent on social media daily (*β* = .11, *p <* .001), accepting stranger’s “friend request” (*β* = .06, *P* = 0.047), FoMO (*β* = .14, *p <* .001) and SMF (*β* = .19, *p <* .001). Furthermore, effect of SUVSM on self-disclosure in social media was mediated by FoMO and SMF.

**Conclusion:**

Inappropriate contents are posted and witnessed by appreciable proportions of nursing students. Positive attitude towards social media may strengthen FoMO and SMF, which may increase self-disclosure in social media in turn.

## 1. Introduction

Advancement of social technologies and the use of social media have greatly impacted learning behavior, academic achievements, professional identity formation and attitudes of university students [1–4]. Social media have become a significant part of young people’s lives, as recent data indicated that 17.4% of the netizens in China are 20~29 years old [5]. In addition, social technologies have radically expanded and transformed how university students communicate about themselves. Social networks, microblogs, moments in WeChat, and instant messaging services provide several features to disclose individual information such as sharing photos, videos, or status updates [6]. Nevertheless, individuals who are engaged in social media excessively potentially risk their privacy, online professionalism, and mental health.

Social media are Internet-based channels that allow users to interact with one another conveniently and selectively and derive value information from user-generated content [7]. User-generated contents often require some form of self-disclosure, namely, the communication of personal information to others online. In the online context, self-disclosure refers to communicate any information about oneself to others through the internet either reflectively or impulsively [8,9]. Self-disclosure has received extensive attention in medical education, due to possible long-term risks in identity thefts, sexual harassments, cyberstalking, commercial or criminal exploitations or professional reputations [10–13]. Nevertheless, much remained unknown about Chinese nursing students.

Chinese nursing students disclose themselves by sharing personal information on social media, such as profile updates, moments in WeChat, vlog, and microblogs updates [14]. Their reputation and credibility for future professionals becomes at risk when excessive self-disclosure and professional activities are mixed up and appear on social media. Privacy of personal information mixed of personal/professional identities are the medical students’ utmost endorsed concerns about social media use [15,16]. Although nursing students held positive attitudes towards social media, personal and professional lives are expected to be separate from one another [14,17]. Attitudes towards the professional and ethical use of social media will have a great impact on nursing reputation management on social media [14,18]. Many educators have argued for the benefits of engaging in social media, but some have concerns about excessive self-disclosure [15,19]. According to De Gagne et al [20], 36.8% of self-identified nurses and nursing students on Twitter generated those 413 uncivil posts, including profanity (32.7%), sexually explicit or suggestive material (9.0%), name-calling (3.4%), and discriminatory remarks against minorities (2.2%). Chinese nursing students may jeopardize their professional credibility by excessively disclosing private information on social media. Given the negative consequences of excessive self-disclosure on professionalism, it could be insightful to identify critical factors affecting self-disclosure and fill the gap between attitudes and self-disclosure in nursing students.

The relationship between self-disclosure and psychological well-being has been found as bidirectional [21]. People low in psychological distress disclose more positively and honest information [22]. Mental health problems concerning social media usage have become more prevalent mainly due to all-time access to online activities [23,24]. Myriad studies have found the problematic social media usage is typically accompanied by a shrinkage of the one’s social circle, an increase in loneliness, depression, fear of missing out (FoMO), social media fatigue (SMF) and online addiction [24–26].

Previous findings also suggested that the effect of attitudes towards social media on self-disclosure could be mediated by FoMO and SMF. For instance, one’s positive-presentation increased the level of online social anxiety [27]. FoMO is a form of social anxiety, acting as a predictor of online risk behaviors among adolescents [24,28]. FoMO enhances individuals’ need to stay connected and communicate with other people, leading to excess self-disclosure online. SMF was found to be associated with self-disclosure in a study [29]. Some studies identified that one’s attention to impression management concern has positive influence on SMF, which is also relates to FoMO [30–33]. Frequent and excessive of experiences of FoMO are associated with negative outcomes, including problem internet use, anxious attachment, gaming disorder, SMF, and social media addiction [23,34]. In accordance with these findings, we proposed a model in this study that is depicted graphically below.

**Fig 1:**
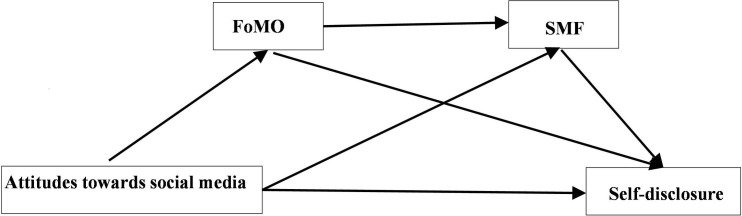
Relationships of attitudes towards social media, FoMO, SMF and self-disclosure.

Although it has been a decade since of social media became popular, little research has focused on the relationship of personal self-disclosure, mental health problems concerning social media use and attitudes towards social media. This study was aimed to investigate Chinese nursing students’ attitudes, personal self-disclosures, mental health problems related to their social media usage, and inappropriate behaviors on social media, and examine the relationship of attitudes towards social media, mental health problems, and personal self-disclosure online, as postulated in Fig 1 in this study.

## 2. Methods

### 2.1 Sample and data collection

A sample of 1054 nursing students were recruited in 7 universities and 4 vocational and technical colleges/schools in China. Participants were approached by the faculty from 11 university/colleges/schools. Participation in the study were entirely based on voluntarism and cooperativeness. Data were collected with structured questionnaires in October, 2020. Participants filled out questionnaires online via Questionnaire Star. The mean age of respondents was 19.51 (SD = 1.73) with a range of 17~32 years. Of the 1054 participants, 13.3% were male and 48.8% lived in urban areas. About three quarters of the responses were undergraduate and graduate nursing students (76.2%), the rest was junior college nursing students.

### 2.2 Instruments

#### Students’ Use and Views of Social Media (SUVSM)

The Chinese version of SUVSM [14] was adapted to measure nursing students’ usage and attitudes towards social media in China. The reliability of the whole scale was high (Cronbach’s α = 0.92). Responses to the twelve questions on attitudes towards using social media were rated from 1 (totally disagree) to 5 (totally agree). Higher scores indicate more positive attitude.

#### Fear of Missing Out Scale (FoMOs)

The Chinese version of this scale was implemented to measure students in China [24]. The FoMOs comprises 12 items with five ratings from 1 (totally disagree) to 5 (totally agree). The reliability of FoMOs was 0.91 (Cronbach’s α) in this study. High scores indicate high level of FoMO.

#### Social media fatigue scale

The scale was referenced to previous studies [25,35–37]. The tool is composed of 18 items articulated in three subscales: anxiety (8 items), information value (4 items), escaping from social media (6 items). Response options for each item ranged from 1 (strongly disagree) to 5 (strongly agree). The Cronbach reliability (α) was respectively 0.93, 0.91 and 0.91 for the three dimensions in this study. High scores indicate a high level of SMF.

#### Self-disclosure in social media

The tool is composed of 21 items in two subscales: (1) Basic information showed on social media (real-time position, social relations, consumption information, personal photos and videos, location, newsletter, education, emotional state, marriage, height and weight, birthday, hobby, gender, and name). (2) Information viewed by others (name, gender, hobby, education, occupation, location and consumption). Responses for each item are rated by five points ranging from 1 (strongly disagree) to 5 (strongly agree) on a Likert scale. The Cronbach’s α was respectively 0.93 and 0.92 for the two dimensions in this study. High scores indicate a high level of self- disclosure on social media.

#### Social media use

Social media use was assessed with the following items: (1) Social media platforms; (2) Number of social media accounts; (3) Number of hours spent on social media (h); (4) Contents preferences on social media; (5) Purposes of updating social feed; (6) Motivations for using social media; (7) People who interact most frequently on social media. (8) Privacy setting on social media platforms.

#### Professionalism towards social media

Professionalism towards social media was assessed with the following items: (1) Strangers’ friend request; (2) Patients’ friend request; (3) Posting photos of the clinical department; (4) Inappropriate contents posted or witnessed on social media; (5) Are you familiar with relevant regulations on the social media usage? (6) Do you have class about clearly defined policies and procedures regarding social media use? In this study, inappropriate behaviors on social media were defined as posting complaints about schoolmates or teachers, complaints about school or workplace, drinking alcohol, patient confidentiality, drug abuse, racial discrimination, sexism, abuse, violence, and sexually suggestive photos.

#### Demographic questionnaire

Demographic information was also collected including the basic information like gender, age, residential setting (rural or urban), education attainment (junior college, undergraduate and above), single-child status (yes or no), and marital status of parents (married, single parent or stepparent, others).

### 2.3 Ethical consideration

This study followed scientific and ethical principles of research. The research protocol was reviewed and approved by the Hubei University of Chinese Medicine Human Ethics Committee (2018-ICE-023). Prior to the collection of data, the purposes and procedures of this study were explained to the participants. They were informed that they would withdraw from the study at any time. Data were collected only from those who voluntarily agreed and provided written consent to participate in the study.

### 2.4 Data analysis/modeling

Descriptive statistics of the participants included demographics, social media use, the scores of SUVSM, FoMO, SMF, professionalism towards social media and self-disclosure in social media. Linear multiple regression models were fitted to identify significant factors (*p* < 0.05) associated with SUVSM, SMF, FoMO, self-disclosure in social media and social media use. The linear multiple regression models are reported in Tables 1 and 2: (1) Model in Table 1 included SMF as the dependent variable and social media use, professionalism towards social media (excluding improper content posted or witnessed), FoMO, SUVSM, and demographic variables as the independent variables. (2) Model in Table 2 specified “self-disclosure in social media” as the dependent and professionalism towards social media (excluding improper content posted or witnessed), social media use, SMF, FoMO, and SUVSM, demographics influencing factors as the independent variables. These analysis and modeling were conducted with the SPSS (v26).

**Table 1:** Determinants of SMF among nursing students (n = 1054).

Variables	B	SE	*β*	*t*	95% CI	*p*
Gender	1.88	1.12	0.05	1.68	-0.32~4.07	0.093
Education	1.60	0.88	0.06	1.82	-0.12~3.31	0.068
**Motivations for Using Social Media**						
To stay up-to-date with news and current events	1.74	0.81	0.07	2.15	0.15~3.32	0.032
To stay in touch with what my friends are doing	2.47	1.14	0.07	2.19	0.26~4.72	0.029
**Purposes of Updating Social Feed**						
Let others know your recent situation	-1.84	0.75	-0.08	-2.44	-3.32~-0.36	0.015
Familiar with relevant regulations on the social media usage	-1.42	0.39	-0.10	-3.57	-2.20~-0.64	< .001
FoMO	0.59	0.04	0.41	13.30	0.50~0.67	< .001
SUVSM	0.23	0.04	0.17	5.36	0.15~0.32	< .001
R^2^	0.33					

Note: SE = standard error.

**Table 2:** Determinants of self-disclosure in social media among nursing students (n = 1054).

Variables	B	SE	*β*	t	95% CI	*p*
Gender	-1.72	1.26	-0.05	-1.36	-4.21~0.76	0.173
Education	3.05	0.99	0.10	3.08	1.10~4.99	0.002
**Purposes of Updating Social Media Feed**						
Sharing moments or Weibo, etc.	2.25	0.86	0.09	2.62	0.57~3.94	0.009
Follow others	-3.33	1.46	-0.07	-2.28	-6.19~-0.47	0.023
**The Most Interacted Groups**						
Netizens	2.15	1.05	0.07	2.05	0.10~4.21	0.040
Strangers	-4.44	1.79	-0.08	-2.48	-7.95~-0.93	0.013
Stranger’s “friend request”	1.59	0.80	0.06	1.99	0.02~3.15	0.047
Time spent on social media daily	1.31	0.36	0.11	3.60	0.59~2.02	< .001
FoMO	0.21	0.05	0.14	3.88	0.10~0.32	< .001
SMF	0.20	0.04	0.19	5.55	0.130~0.27	< .001
R^2^	0.24					

The direct and indirect effects of the path model (Fig 1) were estimated with bootstrapping standard errors using the PRCOESS Macro (3.0) by Hayes [38], specifically, the direct effect of SUVSM on self-disclosure online and the indirect effects of SUVSM on self-disclosure online through SMF and FoMO. Control variables included demographic information, professionalism towards social media, and social media usage. The standard errors of these effects and their 95% bias-corrected Confidence Intervals (CI) were estimated using 5000 bootstrap samples.

## 3. Results

### 3.1 Social media usage

S1 Table shows that 46.0% students had 3~4 social media accounts, and 40.1% of them spent 2~4 h per day using social media. Motivations for using social media were staying in touch with what my friends are doing (88.3%), researching/finding products to buy (75.7%) and finding funny or entertaining contents (68.5%). Regarding the question “Contents preferences on social media”, 75.4% of participants reported useful knowledge information, 75.2% of the participants reported friends’ updates and 65.0% reported entertainments. Regarding the question “The Most Interacted Groups”, 90.1% reported friends, 80.9% reported schoolmates, 18.9% reported net friends, and 5.5% reported strangers.

### 3.2 Mental health problems concerning social media usage

The mean score of FoMO and SMF was 33.17 (SD = 8.50) and 46.78 (SD = 12.26), respectively. SMF was positively associated with “to stay in touch with what my friends are doing”, “to stay up-to-date with news and current events”, FoMO and SUVSW, as indicated by the regression coefficients in Table 1.

### 3.3 Self-disclosure in social media

**Fig 2:**
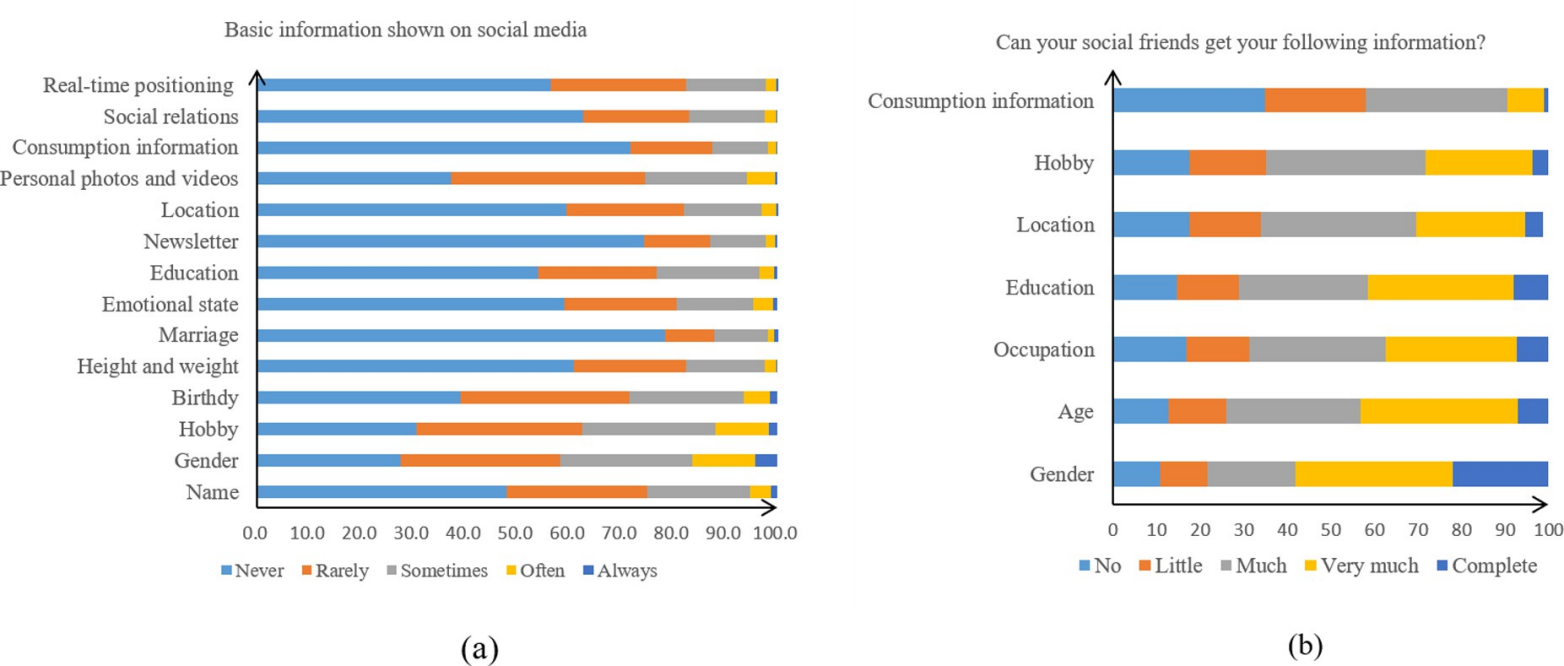
Self-disclosure in social media (n = 1054). a) Basic information shown on social media; b) What information about you can be obtained by your social friends from social media platforms?.

As depicted Fig 2A, a total of 15.3% students reported that they “sometimes” showed real-time positioning on social media, while 5.3% of them indicated they “often” posted photos or videos. Moreover, nearly 2/3 students reported that they did not have privacy setting on sina Weibo and QQ. Regarding the question “can your social friends get your following information online?”, more than 1/3 students reported their net friends could view their following information from their social media accounts: gender, age, occupation, location, and education level (Fig 2B). Table 2 shows that self-disclosure was also associated with education, sharing moments or Weibo, etc., net friends who you interact with most frequently on social media, time spent on social media daily, accepting stranger’s “friend request”, SMF and FoMO.

### 3.4 Professionalism towards social media

About of 48.1% of the respondents reported they were not familiar with relevant regulations on the social media usage. Half of the respondents (50.6%) respondents reported that they had class about clearly defined policies and procedures regarding social media use. 56.7% students claimed that they accepted a stranger’s “friend request”. In addition, 11.1% of respondents accepted a patient’s “friend request”. About 8.8% participants reported they uploaded photos of clinical departments and 17.4% students acknowledged they posted inappropriate content online, including: complaints about schoolmates or teachers (9.9%), complaints about school or medical department (5.8%), patient confidentiality (0.8%), and drinking alcohol (0.9%). In addition, 54.7% of them witnessed improper posts online, including: complaints about schoolmates or teachers (41.8%), complaints about school or medical department (30.5%), verbal abuse (21.7%), violence (12.1%) and patient confidentiality (5.1%). Furthermore, 37.6% of them noticed improper posts from schoolmates or teachers online. These posts included the following: complaints about schoolmates or teachers (26.0%), complaints about school or medical department (19.3%), abuse (8.8%), drinking alcohol (4.6%), violence (3.8%), sexism (2.8%), and patient confidentiality (2.1%).

### 3.5 Relationship of SUVSW, self-disclosure in social media, SMF and FoMO

**Fig 3:**
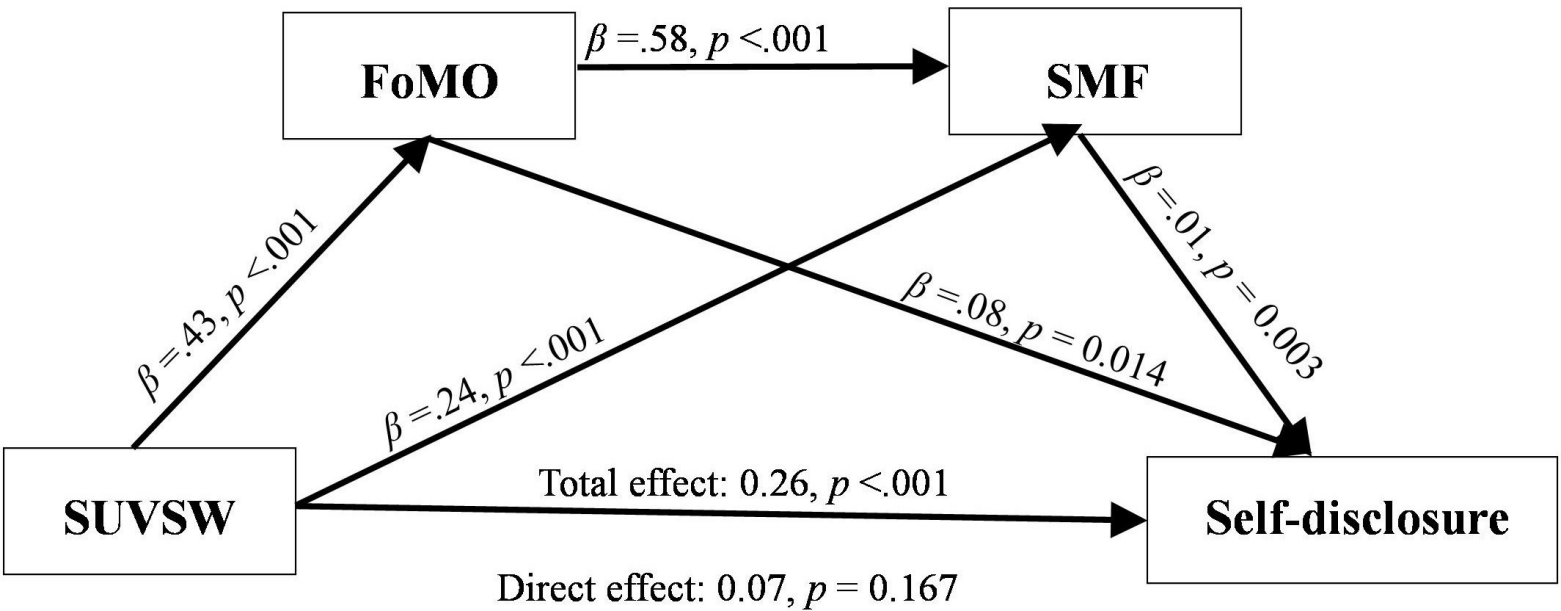
Relationship of SUVSW, self-disclosure in social media, SMF and FoMO.

Table 3 shows the results of indirect effects. Concerning SUVSM, all tested indirect effects were statistically significant, supporting the existence of both simple and mediation effects linking to self-disclosure. As shown in Fig 3, the direct effect of SUVSM on self- disclosure was not statistically significant. The total model accounted for a significant amount of variance in participants’ self-disclosure [R^2^ = 0.27; F _(5.475)_ = 68.00, *p* < .001).

**Table 3:** Indirect effects of SUVSW predicting self-disclosure via FoMO and SMF.

	Estimate	Std Estimate	Lower Bound	Upper Bound
Route of indirect effects				
SUVSM FoMO Self-disclosure	0.10	0.03	0.05	0.15
SUVSM SMF Self-disclosure	0.05	0.01	0.02	0.08
SUVSM FoMO SMF Self-disclosure	0.05	0.01	0.03	0.07
Total indirect effect	0.19	0.03	0.13	0.25

## 4. Discussion

The present study was aimed to investigate the social media usage of Chinese nursing students, including their attitudes, SMF, FoMO, and self-disclosure and the relationship of these constructs. Most of the participants had positive attitudes towards social media, but large proportions of them did not have privacy settings in their social media accounts, posted and viewed inappropriate contents in social media, and were not aware of any regulations on the management of confidential information. Regarding the relationships of these, FoMO and attitudes towards social media positively affected SMF, and the effect of attitudes towards social media on self-disclosure was mediated by SMF and FoMO.

Most students had 3~4 social media accounts and WeChat was the most popular app among nursing students in China in this study. WeChat is the most prevalent mobile app, which has been increasingly utilized as a medium for nursing education [39,40]. Nevertheless, most participants reported that the motivations for using social media were for personal purposes, rather than educational or professional reasons, which is analogous to the findings in other health professions [41,42]. The most interesting contents on social media were useful knowledge information and friends’ updates, answered by most of the participants. During the COVID-19 pandemic, social media have become important sources of useful knowledge information, such as health-related information and protective behaviors [43]. In this study, time spent on using social media daily by most nursing students was in line with other survey [17]. However, 16.2% nursing students spent over six hour per day on social media, higher in percentage than that reported by other studies [17,42]. This discrepancy might be due to sample variations.

The contents posted on social media by nursing students in this study included personal profiles, consumption information, social relations, emotional state, and real-time positioning, however, without privacy protection. Limiting the visibility of personal information to specific people might protect one’s horizontal privacy to a certain extent, but only moments on WeChat and Qzone were installed privacy setting by most nursing students. Over half of students admitted they did not have privacy setting online to limit access to their QQ, sina Weibo, and Tik Tok. This implies that some personal information on their profile that could potentially be shared with be public [10,44]. In addition, approximately 56.7% students claimed that they accepted a stranger’s “friend request”, and 11.1% of participants accepted “friend request” from patients in this survey. Although patients’ “friend request” acceptance rate was relatively lower than previous studies focusing on nurses and surgeons [45,46], blurring between private and professional lives witnessed by patients warrants the attention of health professional communities [13]. It was noteworthy that attitude towards social media was positively associated with self-disclosure. Evidence had shown that individuals with a higher online subjective well-being as a consequence of social media use in terms of positive aspects may engage in more self-disclosure practices [21,47]. Frequent social media use increased the wish to self-disclose online, which was analog to previous study [48]. The decision of whether to self-disclose online is complex and multifarious. In this study, strangers in the groups that participants had most frequently on social media tended to reduced individuals’ self-disclosures, while net friends had opposite effect. A cherishing of private information decreased users’ self-disclosures on the Internet [49].

Inappropriate behaviors on social media undermine the professional images of both the perpetrators and the victims. This study found 37.6% respondents had viewed inappropriate content online from schoolmates or teachers, which was lower than other report [50]. Meanwhile, only 17.4% students reported that they had posted inappropriately contents on social media in this study. To some extent, these numbers echoed previous findings by De Gagne et al. [20]. Although nursing students are expected to develop their professional identity through social media [51]. As students’ physician identity emerges, they are more likely to expect themselves and peers to represent and represent medical professionals in social media [3], nearly half of students in this study admitted they were not familiar with relevant regulations on the management of internet medical and health information and found no classes about usage of social media in their universities. This lacuna causes concerns and calls for actions in nurse educators and university regulators.

We postulated a model about the mediated relationship between the attitudes towards social medial and self-disclosure that was mediated by FoMO and SMF (Fig 1). This model was basically supported by the data of this study. Consistent with previous studies [52,53], SMF was positively related to FoMO, concerns on social relationship [30], and keeping informed about what friends were doing. Due to social media helpfulness, students used social media to stay up-to-date with news and current events, which may contribute to the development of SMF [54]. It is noteworthy that familiarity with relevant regulations on the social media usage could reduce SMF. Familiarity with guidelines for social media usage may also increase individuals’ confidence on social media which could also reduce SMF [55]. Although total indirect effect was significant and appreciable, it accounted for only 26.2% of the total effect. The practical implication is that any intervention on self-disclosure in social media may be best targeted at both the independent variable (the attitudes towards social media) and the mediators (FoMO and SMF), besides other potential factors that were not explored in this study.

## 5. Limitations

This study had some limitations. First, some of the studied variables (e. g. self-disclosure, SUVSW, FoMO and SMF) were based on self-administered questionnaires which may suffer from the negative effects of recall bias and social desirability. Second, the study focused on FoMO and SMF that may mediate relationship of SUVSW and self-disclosure, while omitting other possible mediators, such as problematic social media usage, and perception of professional credibility. Future studies should include these variables. Third, a cross-sectional design did not provide support of the causality among studied variables postulated in this study. Last, the findings from the nursing students’ sample may limit the generalizability of the findings to other majors of college students.

## 6. Conclusion

This study highlights that most nursing students have more than three social media accounts and spend over three hours on social media per day. Appreciable proportions of the participants do not have any privacy settings, are not aware of any regulations on confidentiality of medical information, and tend to mix up their private and professional lives in social media. Some participants post and view inappropriate contents online of their schoolmates and teachers, which may put their careers at risk. SMF was associated with motivations for using social media and updating social feed, familiar with relevant regulations in the social media usage, FoMO and SUVSM. Moreover, self-disclosure in social media was associated with accepting stranger’s “friend request”, time spent on social media daily, FoMO and SMF. Above all, the effects of SUVSW on self-disclosure mediated by SMF and FoMO. Positive attitude toward social media may strengthen FoMO and SMF, which, in turn, may lead to excessive self-disclosure in social media.

## Supporting information

S1 Table (DOCX)

S1 File (XLSX)
