# Supplementary material for: Social media usage of chinese nursing students: Attitudes, motivations, mental health problems, and self-disclosure
Source: PLoS One. 2022 Dec 14;17(12):e0277674. doi: 10.1371/journal.pone.0277674 (PMC9750005; doi:10.1371/journal.pone.0277674)
Supplement: S1 Table — (DOCX) [file pone.0277674.s001.docx]

Table S1 Social media use in nursing students (n = 1054)

| Variables | n | % | Variables | n | % |
| --- | --- | --- | --- | --- | --- |
| **Social media platforms** |  |  | **Number of social media accounts** | | |
| WeChat | 1023 | 97.1 | 0~2 | 265 | 25.1 |
| QQ | 970 | 92.0 | 3~4 | 485 | 46.0 |
| sina Weibo | 588 | 55.8 | 5~6 | 201 | 19.1 |
| Tik Tok | 537 | 51.0 | 7~8 | 49 | 4.7 |
| QQ zone | 436 | 41.4 | 9~ | 54 | 5.1 |
| Little Red Book | 350 | 33.2 | **Time spent on social media (h)** | | |
| **Motivations for using social media** |  |  | 0~2 | 169 | 16.0 |
| To stay in touch with what my friends are doing | 931 | 88.3 | 2~4 | 423 | 40.1 |
| To research/find products to buy | 798 | 75.7 | 4~6 | 291 | 27.6 |
| To find funny or entertaining contents | 722 | 68.5 | 6~8 | 87 | 8.3 |
| Learning | 721 | 68.4 | 8~ | 84 | 8.0 |
| To stay up-to-date with news and current events | 686 | 65.1 | **The Most Interacted Groups** | | |
| To fill up spare time | 642 | 60.9 | Teachers | 132 | 12.5 |
| Playing game | 487 | 46.2 | Parents | 461 | 43.7 |
| To share photos or videos with others | 411 | 39.1 | Relatives | 307 | 29.1 |
| Because a lot of my friends are on them | 178 | 16.9 | Friends | 950 | 90.1 |
| Others | 89 | 8.4 | Schoolmates | 853 | 80.9 |
| **Purposes of updating social feed** |  |  | Netizens | 199 | 18.9 |
| Let others know your recent situation | 483 | 45.8 | Strangers | 58 | 5.5 |
| Express views | 693 | 65.8 | Others | 46 | 4.4 |
| Share your moments or Weibo, etc. | 448 | 42.5 | **Content preferences on social media** | | |
| Follow others | 89 | 8.4 | Friends’ updates | 793 | 75.2 |
| No purposes | 329 | 31.2 | News | 680 | 64.5 |
| Others | 149 | 14.1 | Entertainments | 685 | 65.0 |
| **Privacy setting on social media platforms** |  |  | Services | 377 | 35.8 |
| WeChat | 711 | 67.5 | Knowledge | 795 | 75.4 |
| QQ | 421 | 39.9 | Health information | 460 | 43.6 |
| sina Weibo | 224 | 21.3 | Sports | 136 | 12.9 |
| Tik Tok | 214 | 20.3 | Goods | 210 | 19.9 |
| QQ zone | 696 | 66.0 | Others | 152 | 14.42 |
